# Depression, Anxiety and Symptoms of Stress among Hong Kong Nurses: A Cross-sectional Study

**DOI:** 10.3390/ijerph120911072

**Published:** 2015-09-07

**Authors:** Teris Cheung, Paul S.F. Yip

**Affiliations:** 1 School of Nursing, Hong Kong Polytechnic University, Hong Kong, China; 2 Centre for Suicide Research and Prevention, University of Hong Kong, Hong Kong, China; E-Mail: sfpyip@hku.hk

**Keywords:** anxiety, depression, epidemiology, mental health, nurses, prevalence, stress

## Abstract

Recent epidemiological data suggests 13.3% of Hong Kong residents suffered from Common Mental Disorders, most frequently mixed anxiety and depressive disorder. This study examines the weighted prevalence and associated risk factors of depression, anxiety and stress among Hong Kong nurses. A total of 850 nurses were invited to participate in this cross-sectional study. Participants completed the Depression, Anxiety and Stress Scale 21 and multiple logistic regression was used to determine significant relationships between variables. Chronic past-year illness and poor self-perceived mental health were significant correlates of past-week depression, anxiety and stress. It confirmed further positive correlations between depression and divorce, widowhood and separation, job dissatisfaction, disturbance with colleagues, low physical activity levels and sleep problems. Marital status; general medicine; sleep problems, and a lack of leisure significantly correlated with anxiety. Stress was significantly associated with younger age, clinical inexperience, past-year disturbance with colleagues, low physical activity, no leisure and drinking alcohol. Nurses were more depressed, anxious and stressed than the local general population, with over one-third of our respondents classified as subject to these disorders.

## 1. Introduction

Epidemiological data taken from representative surveys of China as a whole consistently report that psychiatric disorders affect approximately one-third of the population over their lifetime [1,2,3,4,5] In the United States, psychiatric disorders, in particular depression, affect 7% of men and 12% of women annually [6]. A Japanese survey found 25.6% of men and 29.5% of women generally were depressed [7]. 

Chinese figures giving one-month prevalence estimates of mental disorder in 63,004 adults from four provinces suggest a 17.5% incidence of depression (95% CI 16.6–18.5) [5]. A recently published Mental Health Morbidity Survey in Hong Kong (HKMMS) [8] further reveals that, among 5719 Chinese adults aged 16 to 75 years in the general population, a weighted one-week prevalence estimate for Common Mental Disorders (CMD) of 13.3% (95% CI 12.40–14.20). The most frequent diagnoses for individuals in this cohort were mixed anxiety and depressive disorder.

Depression is a common mental disorder in some occupational and unemployment sectors. The projection is for depression to become the second most common cause of disability by 2020 [9,10]. Women are especially at risk of becoming depressed [11,12]. A recent cross-sectional study in China examined the prevalence of depression among 1592 nurses. 61.7% (*n* = 886) had mild depressive symptoms and 25.1% (*n* = 222) moderate to severe symptoms [13]. A similar study in South Korea [14] examined the association between job-related stress, emotive or emotionally arduous work, and depressive symptoms among 441 registered female front-line nurses. Results revealed that approximately 38% of these nurses were experiencing depressive symptoms. Younger or single nurses reported higher levels of depressive symptoms than their married counterparts (OR = 2.88, 95% CI 1.32–6.27). Apart from marital status, being forced to act superficially, rather than sincerely, was the second strongest predictor of depressive symptoms in nurses (OR = 2.46, 95% CI 1.56–3.86); the third strongest was lack of professional reward or recognition (OR = 1.99, 95% CI 1.07–3.70). Superficial behavior in Yoon’s work was defined as “managing the expression of behavior rather than feelings” [15]. The behavior may involve the suppression of true feelings, as possibly necessitated by nurses needing to put on a positive front for the consumers of healthcare services [16]. The hypothesis is that prolonged superficial behavior may cause people to become estranged from their genuine feelings, with adverse effects for their mental wellbeing [14]. Yoon’s findings are consistent with previous studies suggesting the need to put on a front exerts a psychic toll [17,18,19].

Apart from age, marital status, suppressing emotions and feeling unrewarded, research has identified other common risk factors for depression, anxiety and stress [6,7,12]. One is being a woman. Another is being divorced, widowed or separated; substance abuse or being dependent on alcohol; not exercising, and sleeping badly [20]. A family history of mental disorder is also an indicator [8,21].

Despite these findings, little is known on whether female nurses in Hong Kong are at greater risk of psychiatric morbidity than their male counterparts. Traditionally, nursing is a female profession. Might, further, superficial behavior pose an especial risk to nurses’ mental health in East Asian countries?

In Hong Kong, nurses account for over 70% of healthcare personnel [22]. In 2014, there were 48,057 nurses registered with the Hong Kong nursing council, of which 44,379 are registered or enrolled as general nurses and 3668 as mental health nurses [23]. The ratio of general nurses to mental health nurses is approximately 12:1 and the male to female gender ratio is approximately 7:1 [23].

With the HKSAR population growing by over seven million since 2014, nurses have to deal with the increasingly complex care of greater numbers of patients in hospitals. They are often short-staffed and may be poorly rewarded [24,25,26]. They enjoy a low level of autonomy [27], including over their own decision-making; may have to work under severe time pressure with inadequate resources and fractious colleagues [28], and can encounter workplace violence [29,30]. In consequence, nurses are at a high risk of developing work-related stress and associated forms of psychiatric morbidity, including depression [10,25,31,32].

There have been few recent studies of ethnically Chinese nurses’ psychic wellbeing [13]. This may be because of the assumption that nurses’ role in ‘caring for the sick’ requires them to be physically and mentally sound. But nurses’ subjection to persistently high public and professional expectations poses mental health risks [33].

It would not be responsible to claim that nurses are, at all times, physically and mentally healthy without seeking to estimate the prevalence of psychiatric morbidity in the profession. More significantly, it would be easy to dismiss questions of nurses’ psychological well-being on the grounds the evidential base supporting any conclusion is too slender. The primary focus of this study is to estimate the prevalence of depression, anxiety and symptoms of stress, and their associated factors, in the nursing workforce in Hong Kong. More significantly, this is the first-ever prevalence study in the Hong Kong healthcare profession to compare and contrast the associated factors of depression, anxiety and symptoms of stress with previous published works in the nationwide.

## 2. Experimental Section

### 2.1. Aim

This paper forms part of a larger survey-based study of nurses’ mental health. Specifically, it sets out to examine the weighted prevalence of depression, anxiety and stress among nurses in the context of a statement of the socio-demographic characteristics of nurses working in healthcare settings in Hong Kong.

### 2.2. Study Design

This study used a cross-sectional survey design. It took account of existing nursing literature on mental health in drawing up a nine-section, web-based survey, administered by nurses to themselves. This paper only reports weighted estimates for depression, anxiety and symptoms of stress as measured by a short version of Depression Anxiety and Stress Scale (DASS 21) [34], together with related mental health-related components.

### 2.3. Participants

The researchers invited the participation of all member nurses registered with the Association of Hong Kong Nursing Staff (AHKNS), the largest professional organization for Hong Kong nurses. A mass invitation email with a hyperlink was delivered to all members meeting our selection criteria of being aged between 21 and 65, female or male in any clinical specialty, currently employed full-time (44 h) working in any healthcare setting (a private or public hospital, with non-government organizations, or in residential homes or homes for the elderly) and registered with the Hong Kong Nursing Council. We excluded those unable to read Chinese as our version of the Mental Health Survey was in this language.

### 2.4. Ethical Considerations

The study was approved as a social science project by the Human Research Ethics Committee for Non-Clinical Faculties (HRECNCF) and Institutional Review Board of a local university in Hong Kong. Since some survey questions were sensitive, a telephone directory of professional helplines was listed on its last page.

### 2.5. Data Collection Tools and Measurements

Socio-demographic and other work-related information was obtained via a self-reported self-administrative web-based survey. Depression, anxiety and symptoms of stress were measured by Lovibond and Lovibond’s short version of the Depression Anxiety Stress Scale (DASS) [34]. This version, the Depression Anxiety Stress Scale 21 (DASS_21_), has been validated as a reliable self-administered psychological instrument consisting of 21 items in three domains. Each domain comprises seven items assessing three dimensions of mental health symptoms: depression, anxiety and stress. Respondents were required to indicate the presence of these symptom(s) over the past week on a 4-point Likert scale scoring from 0 to 3 (0: did not apply at all over the last week, 1: applied to some degree, or some of the time; 2: applied a considerable degree, or a good part of time; 3: applied very much or most of the time). The more severe the symptoms in each dimension, the higher the subscale scores. This instrument is frequently used in clinical and non-clinical samples [34,35,36,37,38], possessing well-established psychometric properties in reliably measuring depression, anxiety and stress (at a Cronbach’s alpha 0.91, 0.84 and 0.90 respectively). It is also believed capable of differentiating between depression, anxiety and stress [34,39,40,41,42]. Our study used the Chinese validated translation of the DASS_21_ as participants were predominantly ethnically Chinese. Scores from each dimension were summed up and categorized as “normal”, “mild”, “moderate”, “severe” and “extremely severe”, according to the DASS manual [34].

### 2.6. Statistical Analysis

Depression, anxiety and stress scores were categorized into dichotomous responses (yes/no) before being submitted to univariate analysis. Participants with a cut-off score of ≥10 in depression, ≥8 in anxiety and ≥15 in stress dimension were considered as having these disorders as referenced by the DASS manual [34]. Statistical analysis was performed using SPSS Version 23.0 for the Windows platform (SPSS Inc.; Chicago, IL, USA).

To yield representative findings for the Hong Kong nursing population, the study adjusted prevalence estimates by using sampling weights reflecting the size of the population as a whole as suggested by the Hong Kong Nursing Council. Specifically, adjustments were made for gender. Prevalence estimates (%) were presented at 95% confidence intervals (95% CI) calculated from the SE.

Univariate analysis derived mean values, standard deviations (SD), frequencies (n) and proportion percentages (%) from categorical and continuous variables. Bivariate and multivariate analyses then measured the strength of associations between variables and sought to identify significant predictors for the outcomes of interest to the study. All tests were two-tailed with the level of statistical significance defined as *p* < 0.05. Results were presented as odds ratios (ORs) and 95% confidence intervals (95% CI).

## 3. Results and Discussion

A total of 850 participants (female = 745) completed the web-based survey, at a 5.3% response rate.

### 3.1. Socio-demographic, Clinical and Other Characteristics of the Sample Population

The majority of the respondents were female (87.6%, *n* = 745), frontline nurses (87.2%, n = 740). The mean age was between 34 and 44 years old (SD ± 2.79). 55% were married, 43% single and 2% divorced, separated or widowed. 70% had obtained a Bachelor degree or above, earned a monthly household income between HKD 40,000 and 59,000 and were general nurses. Respondents had an average 10–20 years of clinical experience (Table 1).

A total of 70.9% of the respondents worked in a shift rotation pattern. Over half reported some form of disturbance with colleagues in the past year and 45% (*n* = 452) some form of workplace violence. 64% were satisfied with their current job (Table 1).

Less than one quarter of our sample (22.3%) suffered from chronic illness, but a large proportion (87%) could not maintain regular exercise, find time to enjoy entertainment (62%) and were physically inactive (76.5%). Around 35% reported sleep problems. In terms of life events, 4.5% reported at least one life event in the past 12 months while 1% acknowledged a history of psychiatric disorder. Current smokers and alcohol drinkers comprised 1.5% and 24% respectively of the sample (Table 1).

**Table 1:** Frequency distribution of respondents by socio-demographic characteristics and selected variables.

Demographic Characteristics (*n* = 850)	Mean	SD	n	Percentage (%)
Sex				
Male			105	12.4
Female			745	87.6
Age (years)	34–44	2.79		
21–24			77	9.0
25–34			275	32.3
35–44			283	33.3
45–54			186	21.9
55–64			29	3.4
Education level				
Bachelor degree or above			595	69.9
Associate degree			109	12.8
Secondary school (From 4 to 7)			147	17.3
Monthly household income (HKD)	40,000–59,000	2.07		
20,000–39,000			240	28.2
40,000–59,000			312	36.7
≥60,000			298	35.1
Marital status				
Single, never married			361	42.5
Married/cohabitant			467	54.9
Divorced/widowed/separated			22	2.6
Position				
Frontline nurses			740	87.2
Ward managers			109	12.8
Specialty				
General nursing			590	69.4
Mental health nursing			260	30.6
Years of employment	10–20 years 0.82			
<10			402	47.3
≥11			448	52.7
Shift work				
No			247	29.1
Yes			603	70.9
Collegial disturbance				
No			346	40.7
Yes			504	59.3
Workplace violence				
No			471	55.4
Yes			379	44.6
Job satisfaction				
No			308	36.1
Yes			542	63.9
Chronic illness				
No			660	77.7
Yes			90	22.3
Exercise				
No			736	86.6
Yes			114	13.4
Physical activity level				
Inactive			650	76.5
Active			200	23.5
Entertainment				
No			524	61.7
Yes			326	38.3
Maintain 7–8 h sleep 3–4 times a week				
No			547	64.3
Yes			303	35.7
Life events in the past year				
No			812	99.5
Yes			38	4.5
Psychiatric disorder				
No			842	99.0
Yes			8	1.0
Smoking status				
No smoking			838	98.5
Smoked <6 cig./week			9	1.1
Smoked ≥7 cig./week			3	0.4
Current drinker				
No			648	76.3
Yes			202	23.7
Self-perceived physical health				
Poor			510	60.0
Good			340	40.0
Self-perceived mental health				
Poor			369	43.4
Good			481	56.6

n: Frequency. SD: Standard deviations.

#### 3.1.1. Depression and Correlates

Overall, the prevalence of depression, anxiety and symptoms of stress came in at 35.8%, 37.3% and 41.1% respectively. Female, divorced/widowed/separated nurses were more prevalent to report depression, anxiety and symptoms of stress than their male counterparts. Age was found to be insignificant with depression but interestingly, seemed to be significant correlate of anxiety and stress symptoms. Results also showed an inverse relationship between age and depression, anxiety and symptoms of stress. As age increased, depressive, anxiety and stress symptoms decreased (Table 2, Table 3 and Table 4).

On bivariate analysis using binary logistic regression, depression was found to be significantly associated with marital status, clinical specialty, upset with colleagues, workplace violence, job satisfaction, chronic illness, levels of physical activity, entertainment, sleep problems, drinking alcohol, psychiatric disorder and self-perceived physical and mental health. Divorced/widowed/separated nurses were 1.7 times more likely than singles to report depressive symptoms (cOR 1.685, 95% CI 0.71–4.02). General nurses were 2.2 times more likely to report depression than mental health nurses (cOR 2.245, 95% CI 1.62–3.12). Nurses encountering past-year collegial disturbance and workplace violence were 3.2 times and 1.5 times more likely to report depression than those without (cOR 3.165, 95% CI 2.32–4.32 and cOR 1.459, 95% CI 1.10–1.94) respectively. Nurses with chronic past-year illness were two times more likely to experience depressive symptoms than those without (cOR 2.026, 95% CI 1.46–2.81). Physical inactivity, lack of entertainment and not able to maintain 7–8 h sleep 3–4 times/week were also significant correlates of depression (all *p*s < 0.001, cOR ranged from 4.0–0.4). Current drinkers were 1.6 times more likely to report depression than non-drinkers (cOR 1.578, 95% CI 1.143–2.178). Nurses with a history of psychiatric disorder were seven times more likely to report depressive symptoms than those without (cOR 7.145, 95% CI 1.32–38.75). Nurses with poor self-perceived physical and mental health status were also found to be significantly correlated with depressive symptoms (cOR 2.751, 95% CI 2.021–3.744 & cOR 5.745, 95% CI 4.23–7.81 respectively) (Table 2).

**Table 2:** Frequency distribution of respondents by depression status and socio-demographic characteristics and other selected variables.

Variables	Depression Symptoms				*p*	cOR	95% CI	
	No	(%)	Yes	(%)			Lower	Upper
	(n)+		(n)≠					
Sex								
Male *	73	69.5	32	30.5	-	-	-	-
Female	472	63.4	273	36.6	0.211	1.33	0.85	2.06
Age (Years)					0.118			
21–24 *	46	60.5	30	39.5	-	-	-	-
25–34	170	61.8	105	38.2	0.848	0.95	0.57	1.60
35–44	176	62.2	107	37.8	0.810	0.94	0.56	1.58
45–54	128	68.8	58	31.2	0.198	0.70	0.40	1.21
55–64	24	82.8	5	17.2	0.037	0.32	0.11	0.94
Educational Level					0.426			
Bachelor degree/above *	376	63.2	219	36.8	-	-	-	-
Associate degree	69	63.3	40	36.7	0.970	0.992	0.65	1.52
Secondary school (Form 4 to 7)	101	68.7	46.0	31.3	0.196	0.774	0.53	1.14
Marital status					0.001			
Single, never married *	214	59.3	147	40.7	-	-	-	-
Married/cohabitant	322	69.0	145	31.0	0.004	0.656	0.49	0.87
Divorced/widowed/separated	10	45.5	12.0	54.5	0.239	1.685	0.71	4.02
Monthly household income (HKD)					0.058			
20,000–39,000	148	61.7	92.0	38.3	0.060	1.410	0.99	2.02
40,000–59,000	190	60.9	122	39.1	0.026	1.462	1.05	2.04
≥60,000 *	207	69.5	91.0	30.5	-	-	-	-
Specialty								
General nursing	347	58.8	243	41.2	<0.001	2.245	1.62	3.12
Mental health nursing *	198	76.2	62.0	238				
Position								
Staff nurse	471	63.6	269	36.4	0.383	1.210	0.79	1.86
Charge nurse *	74	67.9	35.0	32.1	-	-	-	-
Years of employment								
<10	249	61.9	153	38.1	0.217	1.193	0.90	1.58
≥11 *	296	66.1	152	33.9	-	-	-	-
Shift work								
No *	168	67.7	80.0	32.3	-	-	-	-
Yes	378	62.7	225	37.3	0.158	1.254	0.92	1.72
Job satisfaction								
No	145	47.2	162	52.8	<0.001	3.155	2.35	4.24
Yes *	401	73.8	142	26.2	-	-	-	-
Collegial disturbance								
No *	273	78.9	73.0	21.1	-	-	-	-
Yes	272	54.1	231	45.9	<0.001	3.165	2.32	4.32
Workplace violence								
No *	321	68	151	32.0	-	-	-	-
Yes	225	59.4	154	40.6	0.009	1.459	1.10	1.94
Chronic illness								
No *	448	67.9	212	32.1	<0.001	2.026	1.46	2.81
Yes	97.0	51.1	93.0	48.9	-	-	-	-
Life events in the past year								
No *	24	63.2	14	36.8	-	-	-	-
Yes	521	64.2	291	35.8	0.895	0.955	0.49	1.88
Exercise								
No *	453	61.5	283	28.5	<0.001	0.372	0.23	0.61
Yes	92	81.4	21	18.6	-	-	-	-
Smoking status					0.168			
No smoking *	540	64.5	297	35.5	-	-	-	-
Smoked <6 cig./week	3	33.3	6	66.7	0.060	3.87	0.95	15.80
Smoked >7 cig./week	2	66.7	1	33.3	0.890	0.85	0.09	8.18
Current drinker								
No *	432	66.7	216	33.3	-	-	-	-
Yes	113	55.9	89.0	44.1	0.006	1.578	1.143	2.178
Physical activity level								
Inactive	376	57.8	274	42.2	<0.001	3.998	2.642	6.051
Active *	169	84.5	31.0	15.5	-	-	-	-
Entertainment								
No	291	55.5	233	44.5	<0.001	2.854	2.09	3.91
Yes *	254	78.2	71	21.8	-	-	-	-
Maintain 7–8 h sleep 3–4 times/week								
No	309	56.5	238	43.5	<0.001	0.369	0.27	0.51
Yes *	236	77.9	67	22.1	-	-	-	-
Psychiatric disorder								
No *	544	64.6	298	35.4	0.02	7.145	1.32	38.75
Yes	2	22.2	6	77.8	-	-	-	-
Self-perceived physical health								
Poor	282	55.3	228	44.7	<0.001	2.751	2.021	3.744
Good *	263	77.4	77	22.6	-	-	-	-
Self-perceived mental health								
Poor	157	28.8	213	69.8	<0.001	5.745	4.23	7.81
Good *	389	71.2	92	30.2	-	-	-	-

cOR—Crude odds ratio; * Reference group. + Scores of 0–9 (normal); ≠ Scores of ≥10 (mild, moderate, severe, extremely severe).

#### 3.1.2. Anxiety and correlates

Anxiety correlated with both individual and work-related factors. Individual factors included age, marital status, monthly household income, chronic illness, lack of exercise and entertainment, low physical activity, sleep problems, psychiatric disorders and self-perceived health. Work-related risk factors included clinical specialty, years employed, job satisfaction, upset with colleagues and workplace violence (Table 3). Nurses with a monthly household income between HKD 20,000 and 39,000 were 1.9 times more likely than to report anxiety than those earning higher monthly household income (*i.e.*, ≥HKD 60,000) (cOR 1.869, 95% CI 1.31–2.66). A history of chronic illness in the past year and physical inactivity were significantly correlated with anxiety symptoms (cOR 1.811, 95% CI 1.31–2.51 and cOR 2.658, 95% CI 1.833–3.854 respectively). Nurses reporting a lack of exercise, entertainment and unable to maintain 7–8 h sleep 3–4 times a week were 2 to 3 times more likely to report anxiety than those whom were capable to maintain a healthy lifestyle. Nurses with a history of psychiatric disorder were 4.7 times more likely to report anxiety than those without. Those who had poor self-perceived physical and mental health status were 2.9 times and 4.3 times more likely to experience anxiety symptoms than those reporting good physical and mental health (Table 3).

**Table 3:** Frequency distribution of respondents by anxiety status and socio-demographic characteristics and other selected variables.

Variables	Anxiety Symptoms				*p*	cOR	95% CI	
	No	(%)	Yes	(%)			Lower	Upper
	(n)+		(n)≠					
Sex								
Male *	73	69.7	32	30.3	-	-	-	-
Female	460	61.8	285	38.2	0.118	1.422	0.92	2.21
Age					0.008			
21–24 *	45	59.2	31	40.8	-	-	-	-
25–34	153	55.6	122	44.4	0.565	1.163	0.70	1.95
35–44	100	64.7	100	35.3	0.397	0.800	0.48	1.34
45–54	128	68.8	58	31.2	0.151	0.668	0.39	1.16
55–64	24	82.8	5	17.2	0.032	0.315	0.11	0.91
Educational level					0.638			
Bachelor degree or above	378	63.5	217	36.5	-	-	-	-
Associate degree	64	58.7	45	41.3	0.345	1.223	0.81	1.86
Secondary school (Form 4 to 7)	91	62.3	55	37.7	0.794	1.051	0.72	1.53
Marital status					0.000			
Single, never married *	202	55.8	160	44.2	-	-	-	-
Married/cohabitant	320	68.5	147	31.5	0.000	0.578	0.44	0.77
Divorced/widowed/separated	11	50.0	11	50.0	0.684	1.197	0.50	2.85
Monthly household income (HKD)					0.002			
20,000–39,000	130	54.4	109	45.6	0.001	1.869	1.31	2.66
40,000–59,000	197	63.1	115	36.9	0.138	1.290	0.92	1.81
≥60,000 *	206	68.9	93	31.1	-	-	-	-
Specialty								
General nursing	335	56.8	255	43.2	0.000	2.454	1.77	3.41
Mental health nursing *	198	76.2	62	23.8	-	-	-	-
Position								
Frontline nurses	459	61.9	282	38.1	0.238	1.294	0.84	1.98
Ward managers *	74	67.9	35	32.1	-	-	-	-
Years of employment								
<10	230	57.4	171	42.6	0.002	1.549	1.17	2.05
≥11 *	303	67.6	145	32.4	-	-	-	-
Shift work								
No *	166	67.2	81	32.8	0.096	1.303	0.95	1.78
Yes	368	61.0	235	39.0	-	-	-	-
Job satisfaction								
No	160	52.1	147	47.9	0.000	2.027	1.52	2.70
Yes *	373	68.7	170	31.3	-	-	-	-
Collegial disturbance								
No *	274	79.0	73	21.0	-	-	-	-
Yes	260	51.6	544	48.4	0.000	3.540	2.591	4.84
Workplace violence								
No *	318	67.5	153	32.5	-	-	-	-
Yes	216	57.0	163	43.0	0.002	1.570	1.19	2.08
Chronic illness								
No *	98	51.6	92	48.4	-	-	-	-
Yes	435	65.9	225	34.1	0.000	1.811	1.31	2.51
Life events in the past year								
No *	506	62.3	306	37.7	-	-	-	-
Yes	27	71.1	11	28.9	0.310	0.691	0.339	1.410
Exercise								
No	445	60.5	291	39.5	-	-	-	-
Yes *	88	77.2	26	22.8	0.001	2.21	1.39	3.51
Smoking					0.747			
No smoking *	526	62.8	311	37.2	-	-	-	-
Smoked <6 cig. a week	5	55.6	4	44.4	0.462	1.637	0.44	6.09
Smoked ≥7 cig. a week	2	66.7	1	33.3	0.841	0.794	0.08	7.61
Current drinker								
No *	416	64.2	232	35.8	-	-	-	-
Yes	117	57.9	85	42.1	0.102	1.308	0.948	1.806
Physical activity level								
Inactive	376	57.8	274	42.2	0.000	2.658	1.833	3.854
Active *	157	78.5	43	21.5	-	-	-	-
Entertainment								
No	279	53.2	245	46.8	0.000	3.129	2.29	4.28
Yes *	254	78.2	71	21.8	-	-	-	-
Maintain 7–8 h sleep 3–4 times a week								
No	250	45.7	297	54.3	0.000	2.960	2.151	4.072
Psychiatric disorder								
No *	531	63.1	311	36.9	-	-	-	-
Yes	2	25.0	6	75.0	0.048	4.729	1.011	22.11
Self-perceived physical health								
Poor	273	53.5	237	46.5	0.000	2.854	2.10	3.87
Good *	261	76.8	79	23.2	-	-	-	-
Self-perceived mental health								
Poor	164	44.2	206	55.8	0.000	4.25	3.16	5.70
Good *	370	77.1	110	22.9	-	-	-	-

cOR—Crude odds ratio; * Reference group. + Scores of 0–7 (normal). ≠ Scores of ≥8 (mild, moderate, severe, extremely severe).

#### 3.1.3. Stress and Correlates

Stress was significantly associated with age, marital status, monthly household income, clinical specialty, job position and years employed. Front-line general nurses with clinical experience of less than 10 years; who worked shifts; who were unhappy at work; and who had experienced upset with colleagues and faced workplace violence or chronic past-year illness were more likely to report stress (cOR ranged from 0.54 to 3.79). This also held for nurses who did not exercise or take time out to watch entertainment, who drank, had trouble sleeping and saw themselves as suffering from poor physical and mental health (cOR ranged from 1.82 to 5.90) (Table 4).

**Table 4:** Frequency distribution of respondents by stress status and socio-demographic characteristics and other selected variables.

Variables	Stress Symptoms				*p*	cOR	95% CI	
	No	(%)	Yes	(%)			Lower	Upper
	(n)+		(n)≠					
Sex								
Male *	70	66.3	35	33.7	0.102	1.431	0.91	2.20
Female	431	57.9	314	42.1	-	-	-	-
Age					0.000			
21–24 *	39	50.6	38	49.4	-	-	-	-
25–34	131	47.6	144	52.4	0.637	1.130	0.681	1.88
35–44	180	63.4	104	36.6	0.043	0.591	0.36	0.98
45–54	126	67.4	61	32.6	0.011	0.493	0.29	0.85
55–64	26	89.7	3	10.3	0.001	0.109	0.03	0.41
Education level					0.306			
Bachelor degree or above *	350	58.8	245	41.2	-	-	-	-
Associate degree	58	53.7	50	46.3	0.308	1.239	0.82	1.87
Secondary school (Form 4 to 7)	93	63.3	54	36.7	0.338	0.833	0.574	1.21
Marital status					0.000			
Single, never married *	181	50.1	180	49.9	-	-	-	-
Married/cohabitant	307	65.7	160	34.3	0.000	0.526	0.40	0.70
Divorced/widowed/separated	12	57.1	9	42.9	0.574	0.779	0.325	1.87
Monthly household income (HKD)					0.000			
20,000–39,000	116	48.5	123	51.5	0.000	2.019	1.43	2.86
40,000–59,000	189	60.6	123	39.4	0.201	1.240	0.89	1.72
≥60,000 *	196	65.6	103	34.4	-	-	-	-
Specialty								
General Nursing	316	53.5	275	46.5	0.000	2.161	1.58	2.96
Mental health nursing	185	71.2	75	28.8	-	-	-	-
Position								
Frontline nurses	427	57.6	314	42.4	0.052	1.525	0.10	2.33
Ward managers *	74	67.3	36	32.7	-	-	-	-
Years of employment								
<10	213	53.0	189	74.0	0.001	1.589	1.21	2.09
≥11	288	64.3	160	35.7	-	-	-	-
Shift work								
No *	162	65.6	85	34.4	-	-	-	-
Yes	339	56.2	264	43.8	0.012	1.480	1.09	2.01
Job satisfaction								
No	136	44.3	171	55.7	0.000	2.560	1.92	3.41
Yes *	364	67.2	178	32.8	-	-	-	-
Collegial disturbance								
No *	266	76.9	80	23.1	-	-	-	-
Yes	235	46.6	269	53.4	0.000	3.785	2.791	5.134
Workplace violence								
No *	294	58.7	207	41.3	-	-	-	-
Yes	177	50.7	172	49.3	0.023	1.376	1.045	1.812
Chronic illness								
No *	90	47.4	100	52.6	-	-	-	-
Yes	411	62.3	249	37.7	0.000	0.543	0.392	0.751
Life events in the past year								
No *	477	58.7	335	41.3	-	-	-	-
Yes	23	62.2	14	37.8	0.714	0.882	0.451	1.724
Exercise								
No	418	56.7	319	43.3	0.001	2.088	1.344	3.24
Yes	83	73.5	30	26.5	-	-	-	-
Smoking					0.450			
No smoking *	495	59.1	342	40.9	-	-	-	-
Smoked< 6 cig. a week	3	33.3	6	66.7	0.224	2.308	0.60	8.89
Smoked ≥7 cig. a week	2	66.7	1	33.3	0.737	0.678	0.07	6.51
Current drinker								
No *	405	62.4	244	37.6	-	-	-	-
Yes	96	47.5	106	52.5	0.000	1.822	1.33	2.51
Physical activity level								
Inactive	341	52.5	309	47.5	0.000	3.584	2.456	5.231
Active *	160	80.0	40	20.0	-	-	-	-
Entertainment								
No	261	49.7	264	50.3	0.000	2.847	2.11	3.84
Yes *	240	73.8	85	26.2	-	-	-	-
Maintain 7–8 h sleep 3–4 times a week								
No	279	51.0	268	49.0	0.000	2.638	1.945	3.578
Yes *	222	73.3	81	26.7	-	-	-	-
Psychiatric disorder								
No *	499	59.3	343	40.7	-	-	-	-
Yes	2	25.0	6	75.0	0.077	4.022	0.860	18.80
Self-perceived physical health								
Poor	247	48.4	263	51.6	0.000	3.151	2.34	4.25
Good *	254	74.7	86	25.3	-	-	-	-
Self-perceived mental health								
Poor	132	35.8	237	64.2	0.000	5.908	4.38	7.98
Good *	369	76.7	112	23.3	-	-	-	-

cOR—Crude odds ratio; * Reference group. + Scores of 0–14 (normal). ≠ Scores of ≥15 (mild, moderate, severe, extremely severe).

All the independent variables considered are potentially influential on the outcome based on of our extensive literature review. Variables registering a *p* value of <0.25 in the bivariate analysis were taken by the study as important risk factors for depression, anxiety and stress and were entered on this basis into multivariate logistic regression. The cutoff point (*p* < 0.25) for selecting candidate variables for multivariable logistic regression was chosen on the basis of Hosmer and Lemeshow’s recommendation so as to avoid omission of potentially important covariates that fail to be significant in univariate analysis. At the same time, this cutoff can be used to screen out those variables of questionable importance [43]. A forward likelihood ratio (LR) method was used to predict the associated variables for depression, anxiety and stress in three separate models. 

#### 3.1.4. Multivariate Analyses

In the final model, seven variables—marital status, chronic illness, job satisfaction, upset with colleagues, levels of physical activity, sleep problems, and self-perceived mental health—emerged as significant correlates of depression (Table 5). The strongest correlate was self-perceived mental health, which had an adjusted odds ratio (aOR) of 3.5 times, followed by physical activity (aOR 2.4) and run-ins with colleagues (aOR 1.9). Divorced, separated or widowed and married individuals were 2.3 times and 0.7 times respectively more likely than singles to have depressive symptoms. Individuals dissatisfied with their current jobs were 1.6 times more likely than those satisfied. Those with a history of chronic illness in the past year were 0.7 times more likely than those without to report symptoms. Respondents unable to sleep some 7-8 hours a night sleep at least 3 to 4 times per week were 1.5 times more likely to have depressive symptoms than those without sleeping disruptions.

For anxiety (Table 5), marital status, clinical specialty, chronic illness, “entertainment”, sleep problems and self-perceived mental health remained significant predictors (Table 5) in the final model. Anxiety was 2.5 times more likely in respondents reporting poor self-perceived mental health, 1.7 times more likely in individuals unable to sleep 7–8 h 3–4 times per week and 1.6 times more likely found in general than in mental health nurses. Individuals not finding time for entertainment but with a history of chronic illness were 2.1 times and 0.7 times more likely to experience anxiety symptoms. Divorced, widowed or separated respondents, first, and married respondents, second, were 1.8 times and 0.7 times more likely than singles to report anxiety symptoms.

Poor self-perceived mental health was the strongest predictor of stress (Table 5), registering an OR of 3.9 (95% CI 2.77–5.37), followed by disturbance with colleagues (aOR 2.6). Individuals who were physically inactive and drank alcohol 1–2 times per month were 1.8 times more likely to report symptoms of stress than physically active non-drinkers. Individuals not finding time for entertainment and with a history of chronic illness were 1.6 times and 0.6 times more likely to report stress than those free of chronic illness and who found time to relax at least once a week. Age and clinical experience were also significant correlates of stress, with differences in age ranging from aOR 1.2 to 0.1. The younger respondents were, the more likely to report symptoms of stress.

Nurses with less than 10 years of clinical experience were 0.5 times more likely to report symptoms of stress than those with over 11 years’ experience. There was also a significant correlation between depression, anxiety and symptoms of stress (all *p*s < 0.001, two-tailed; r = 0.692 for depression and anxiety, r = 0.732 for depression and anxiety, r = 0.725 for anxiety and stress).

**Table 5:** Multiple logistic regression model predicting depression, anxiety and stress symptoms among Hong Kong nurses.

Variable	Categories	B	S.E.	Wald	*p*-Value	aOR	95% CI	
							Lower	Upper
Depression								
Constant		−2.297	0.319	51.785	0.000			
Marital status				7.201	0.027			
	Single, never married *	-	-	-	-	-	-	-
	Married/cohabitant	−0.310	0.167	3.432	0.064	0.734	0.53	1.02
	Divorced/widowed/separated	0.836	0.531	2.479	0.115	2.306	0.82	6.53
Chronic illness	No *	-	-	-	-	-	-	-
	Yes	−0.428	0.191	4.993	0.025	0.652	0.45	0.95
Job satisfaction	No	0.469	0.173	7.337	0.007	1.598	1.14	2.24
	Yes *	-	-	-	-	-	-	-
Collegial disturbance	No *	-	-	-	-	-	-	-
	Yes	0.623	0.179	12.118	0.000	1.865	1.31	2.65
Physical activity level	Inactive	0.869	0.235	13.707	0.000	2.383	1.51	3.78
	Active *	-	-	-	-	-	-	-
Maintain 7–8 h sleep 3–4 times per week	No	0.408	0.188	4.706	0.030	1.504	1.04	2.17
	Yes *	-	-	-	-	-	-	-
Self-perceived mental health	Poor	1.249	0.172	52.538	0.000	3.487	2.49	4.89
	Good *	-	-	-	-	-	-	-
Anxiety								
Constant		−2.179	0.306	50.748	0.000			
Marital status				9.443	0.009			
	Single, never married *	-	-	-	-	-	-	-
	Married/cohabitant	−0.437	0.164	7.098	0.008	0.646	0.47	0.89
	Divorced/widowed/separated	0.558	0.529	1.115	0.291	1.748	0.62	4.93
Specialty	General nursing	0.471	0.188	6.288	0.012	1.602	1.11	2.32
	Mental health nursing *	-	-	-	-	-	-	-
Chronic illness	No *	-	-	-	-	-	-	-
	Yes	−0.370	0.189	3.850	0.050	0.691	0.48	1.00
Entertainment	No	0.736	0.182	16.428	0.000	2.087	1.46	2.98
	Yes *	-	-	-	-	-	-	-
Maintain 7–8 h sleep 3–4 times per week	No	0.528	0.185	8.121	0.004	1.695	1.18	2.44
	Yes *	-	-	-	-	-	-	-
Self-perceived mental health	Poor	0.909	0.167	29.756	0.000	2.481	1.79	3.44
	Good *	-	-	-	-	-	-	-
Stress								
Constant		−1.207	0.477	6.403	0.011			
Age				21.485	0.000			
	21–24 *	-	-	-	-	-	-	-
	25–34	0.158	0.300	0.277	0.599	1.171	0.65	2.11
	35–44	−0.987	0.388	6.456	0.011	0.373	0.174	0.798
	45–54	−1.173	0.415	7.979	0.005	0.309	0.137	0.698
	55–64	−2.270	0.759	8.945	0.003	0.103	0.023	0.457
Clinical experience	≤10 years	−0.628	0.288	4.776	0.029	0.533	0.30	0.94
	≥11 years	-	-	-	-	-	-	-
Collegial disturbance	No *	-	-	-	-	-	-	-
	Yes	0.946	0.176	28.994	0.000	2.575	1.83	3.63
Chronic illness	No *	-	-	-	-	-	-	-
	Yes	−0.503	0.199	6.363	0.012	0.605	0.41	0.89
Physical activity level	Inactive	0.582	0.247	5.570	0.018	1.790	1.10	2.90
	Active *	-	-	-	-	-	-	-
Entertainment	No	0.437	0.203	4.640	0.031	1.549	1.04	2.31
	Yes *	-	-	-	-	-	-	-
Current drinker	No *	-	-	-	-	-	-	-
	Yes, 1–2 times per month	0.586	0.212	7.641	0.006	1.797	1.19	2.72
	Yes, daily to few times per week	0.488	0.393	1.543	0.214	1.629	0.75	3.52
Self-perceived mental health	Poor	1.350	0.169	64.16	0.000	3.857	2.77	5.37
	Good *	-	-	-	-	-	-	-

aOR—Adjusted odds ratio; * Reference group.

### 3.2. Discussion

Our study revealed a prevalence of depression, anxiety and stress at 35.8%, 37.3% and 41.1% respectively among nurses in Hong Kong. The proportion of nurses suffering from depression, anxiety and symptoms of stress is alarming. These findings for depression and anxiety are almost three times higher than in a recently published large scale Mental Morbidity Survey [8] with a cohort of 5719 Chinese adults aged 16–75 years taken from the general Hong Kong population. Using the Chinese Revised Clinical Interview Schedule (CIS-R), the weighted prevalence estimate for past-week Common Mental Disorders (CMD) was 13.3% (95% CI 12.40–14.20). Respondents with depressive episode and general anxiety disorders (GAD) respectively accounted for 2.9% (95% CI 2.47–3.31) and 4.2% (95% CI 3.70–4.74) of Lam’s sample [8]. Lam’s study also revealed that being a woman, divorce or separation, alcohol misuse and lack of regular exercise predicted depression. Our findings, however, did not report any significant gender differential in these prevalence estimates. Our results do, though, confirm that divorced, widowed or separated individuals report more depression and anxiety than married couples or single individuals (or those who never married). Research has recognized that having a spouse, or partner, in a stable relationship brings a measure of emotional stability, with the power significantly to lower odds of the psychiatric morbidity, including depressive symptoms [20,21,44,45]. Single nurses, who may be fully committed psychologically to their jobs, may be more liable to depression, anxiety and stress, reducing any gender gap in the incidence of these phenomena.

Lam’s study, in common with research on Western countries, also highlights the importance of lifestyle elements in promoting good mental health [21,46]. Our study echoed Lam’s in finding physically inactive respondents at higher odds of reporting depression and symptoms of stress than those physically active. Those not finding time for recreation at once a week were more likely to experience anxiety and symptoms of stress than those who did.

It is noteworthy that chronic illness in our study turned out to be a significant correlate of depression, anxiety and stress. These findings concur with other chronic disease models positing an association between chronic illness and psychiatric morbidity [21,47]. Recent empirical findings also support the notion that better general health may lead to better mental health [20], a claim borne out by our finding that individuals with chronic illness and poor self-perceived mental health were more likely to report depression, anxiety and symptoms of stress. Bivariate correlations also showed self-perceived physical health among our sample to be significantly correlated with self-reported mental health (r = 0.515, *p* < 0.001, two-tailed).

Alarmingly, nearly 65% of our respondents were unable to keep up sleeping for 7–8 h 3–4 nights a week. Are these sleep disruptions owing to work-related factors or factors pertaining to respondents’ personal circumstances? Our results showed that respondents with sleep problems were 1.5 times (OR = 1.5, 95% CI 1.04–2.17) and 1.7 times (OR = 1.7, 95% CI 1.18–2.44) more likely to experience depression and anxiety respectively than those without.

Some authors [7] suggest various symptoms of sleep problems point to depression. It is assumed that depression causes sleep disturbances, although causation (as acknowledged by some) could work the other way, with disturbances being considered a risk factor for depression [48,49,50,51,52]. Sleep disturbances and depression could alternatively be in a mutual cause-and-effect relationship.

It is not uncommon for nurses to suffer from sleep problems. For example, Perry *et al.* [20] cross-sectional study on 382 nurses in Australia showed that approximately 70% (*n* = 268) of nurses reported sleep problems and, further, that sleep problems were a significant predictor of mental health. Our findings accord closely with Perry’s, with nearly 65% (*n* = 547) of our sample reporting sleep problems, of whom 44% (n = 238) reported depressive symptoms. Sleep problems were also a significant correlate of depression and anxiety in our multivariate analysis.

The severity of depression, anxiety and symptoms of stress among respondents was measured by a self-report Depression Anxiety Stress Scale 21 (DASS21) limited to events in the past week. Respondents’ report may thus be subject to under/over-reporting [21], especially if they had experienced negative life events within the study period. DASS 21 is a screening tool but not a diagnostic instrument for psychiatric disorders. It is debatable whether the severity of psychiatric symptoms among our respondents always corresponds to a psychiatric diagnosis, in individual cases, of major depressive or anxiety disorders, or to some form of mixed anxiety and depression disorder (MADD) warranting immediate psychiatric treatment.

Our study was limited by its cross-sectional design, meaning that it could not investigate qualitative or narrative comments concerning respondents’ sleep problems. Shift work rotation and work-related stress may feature as risk factors contributing to nurses’ sleep problems. Much research on nursing recognizes that nurses doing shift work often experience poorer sleep quality [53,54,55,56] and quantity [57]. Prolonged sleep deprivation can lead to disorder in circadian rhythms [58], as shift work runs down individuals’ physical and psychological well-being [59,60,61]. It may also tend to lower nurses’ job performance, jeopardizing patient care and treatment outcomes [62].

A local researcher [55] has investigated the impact of shift work rotation on Hong Kong nurses, concluding that 70% (*n* = 163) of his respondents suffered from poor sleep. A similar Asian study conducted by Lin *et al.* [54] on 266 Taiwan nurses emphasized that job stress was inversely related to sleep quality, while stress was directly correlated with worse self-perceived health. In other words, causal links may hold between shift work rotation, sleep quality, job stress, self-perceived physical and mental health status [54].

Caution needs to be taken in comparing our and Chan’s and Lin’s findings, in that they rely upon both objective and subjective forms of measurement of sleep quality while we used only respondents’ self-reported measurement of average hours of sleep on a weekly basis. Sleep sufficiency may vary among individuals according, amongst other factors, to genetic influence, gender, age, physical environment and other socio-cultural inputs. In our study, however, shift work did emerge as a significant correlate in bivariate analysis in predicting anxiety and levels of stress (*p* = 0.158, OR = 1.25, 95% CI 0.92–1.72 for anxiety and *p* = 0.012, OR = 1.48, 95% CI 1.09–2.01 for stress). Shift work then dropped out of being a significant correlate in multivariate analysis. Therefore a cross-national comparison of findings is recommended to build up a greater body of empirical evidence to found any inference on the negative impact of shift work on nurses.

Although nursing has by nature to provide a 24-h service to patients in hospitals, healthcare providers may consider individual nurses’ diurnal preference (morningness/eveningness) before designating individuals to shifts [63]. A forward (or clockwise) rotating shift pattern (*i.e.*, morning, afternoon and night shifts) may be a good choice of rotation [64]. Nevertheless, nurse managers should assign nurses no more than 5 to 7 working days in a row and grant an adequate rest time between a change of shifts [64]. If nurses are mandatorily required to comply with rotations, they may then build up shift work tolerance (SWT) to mitigate the negative impact of shift work [65]. SWT can be achieved by adopting a healthy lifestyle (*i.e*. regular exercise, balanced diet, adequate rest and sleep) that keeps in place a social support network [66] to reduce nurses’ isolation.

One especially noteworthy feature of the results was the prominence of clinical specialty as a correlate of depression, anxiety and stress in the bivariate analysis. Clinical specialty remained significantly correlated with nurses’ level of anxiety in multivariate analysis. General nurses were 1.6 times more likely than mental health nurses to report anxiety symptoms (*p* = 0.012, OR = 1.60, 95% CI 1.11–2.32). Almost 70% of respondents were general nurses and the proportion of general nurses outnumbered mental health nurses. Since we were not authorized to access the local nursing population other than through the data collection services of a local nursing association, it was impossible to obtain a representative sample through stratified random sampling. Even so, we successfully recruited 260 mental health nurses to our study, a large enough cohort to form a comparison group with general nurses and so obviate any adjustment to the prevalence estimates.

Few works compare levels of depression, anxiety and symptoms of stress among nurse-practitioners of different clinical specialties, meaning there is no current consensus on whether general nurses are more vulnerable to psychiatric morbidity than mental health nurses. Older research [67], which specifically measures the level of stress and coping among general mid-wives and mental health nurses, concluded that, of 500 nurses participants in their study, pediatric nurses within the general stream (acute, community, obstetric and critical care, operation theatre) reported the highest level of stress. Others find intensive care unit (ICU) nurses more stressed than those working in other specialties [68].

Participants in our studies came from too many general clinical sub-specialties to be meaningfully categorized into distinctions yielding statistical significance. We therefore only used one division of clinical specialty (general nurses as against mental health nurses) to compare depression, anxiety and stress levels among respondents. Interestingly, clinical specialty turned out to be a significant predictor of anxiety but not depression or stress in our study. We can only suggest that, statistically speaking, general nurses tend to report anxiety symptoms more frequently than mental health nurses. Nevertheless, a recent study conducted by Wang *et al.* [69] argued that mental health nurses were also vulnerable to depressive symptoms, finding that work stress represents a significant correlate of depression. Wang does not consider general nurses, ruling out comparison between general and mental health stream practitioners. Nevertheless, his findings provide a significant indication that, regardless of clinical specialty and background training, nurses are not immune to mental health issues.

Drawing on Callaghan’s, Lau’s, Wang’s and our findings, we suggest clinical specialty might well be a predicting variable for psychiatric symptoms in nurses. Future studies are required to determine whether our results are replicable concerning the association between clinical specialty and psychiatric symptoms in nursing.

It is generally assumed that job-related stress originates within work environments. Stress may, though, have more external sources. In one case, during the 2003 outbreak of the SARS epidemic in Hong Kong, many healthcare workers found themselves under significant levels of anxiety and stress in treating an elevated influx of new patients in various hospitals. Nurses were physically, psychologically and emotionally tested in having to deal with sufferers throughout the outbreak. Professionalism may require nurses to suppress any show of their emotions in the workplace to maintain an appearance of decorum and uphold the profession’s image. Nurses’ training is to make patients feel safe by suppressing any sign of uncertainty over the outcome of treatment, doubt over whether treatment is optimal or rationally chosen, and any feelings of personal distress [70]. Nevertheless, this burden of emotional labor borne by nurses may make their job more stressful; it may also make them less healthy, more estranged from their feelings and job and more subject to depressive episodes [18,71,72].

Internal job stress may stem from nurses feeling they have too heavy a workload [67], are badly treated by colleagues or not supported by managers. They may be unhappy with their work ambience, feel badly rewarded [14], or hampered in their promotion prospects [73]. They may fear or have suffered violence at the hands of patients or other healthcare workers, or be put on uncongenial rotas [65], be denied power over their own decision-making [73] or have other restrictions placed on their autonomy [69].

Job satisfaction and collegial disturbance were significant correlates of depression and stress in the study’s multivariate analysis, with both variables being positively correlated (r = 0.27, *p* < 0.001, two-tailed). Nearly one and a half decades ago, Lee [74] examined the job satisfaction and autonomy of 190 registered hospital nurses in Hong Kong through a cross-sectional survey (The Index of Work Satisfaction (IWS)) designed to assess attitudes to six components of job satisfaction (autonomy, professional status, pay, interaction, task requirements, organizational policies). Results showed that nurses valued autonomy, professional status and pay as the most important contributors to job satisfaction. A vast majority (87%) of our respondents were frontline nurses responsible for varied clinical duties, including patients’ bed-side care and communication with patients, relatives and other medical professionals. Compared to managerial staff, front-line nurses more frequently bore the brunt of workplace conflicts, sometimes being subject to bullying or emotional abuse at work. Nevertheless, it remains likely that embarrassment and the desire to head off tension causes nurses to under-report emotional abuse to clinical managers, selectively reporting only serious incidents [75]. Existing studies tend to concentrate on incidents originating with patients, downplaying or simply not addressing the impact of workplace conflicts on nurses’ mental well-being [76,77].

Nursing has traditionally been portrayed as a physically and emotionally demanding profession [20,73] that is intolerant of error. Slips (e.g., medication errors; patient falls, cases of negligence) may endanger patient safety and incur costs, lawsuits and public outcry. High public expectations and punishing organizational cultures can give rise, for nurses, to workplace conflicts, which may be exacerbated when nurses find themselves up against stressful time constraints, staff shortages, and limited resources. Nursing teams are typically characterized by a skill-mix, *i.e.*, with nurses of different age groups, years of clinical experience, specialty training working on the same ward or unit. Every registered frontline nurse is professionally trained to be independent and competent in various nursing tasks—a skillset forming the basis of that individual’s legal, ethical, moral and professional liability for any medication errors they make or patient incidents they are taken to cause. As a result, front-line nurses may show zero tolerance to incompetent or inexperienced colleagues. Some nurses may also be reluctant to offer a helping hand to others, so reducing the risk of being blamed for others’ mistakes—and of risking their own license to practice. Conflicts of interest may escalate into disturbance between colleagues, impacting on staff morale, team spirit and job satisfaction.

Historically, nurses have limited workplace autonomy, with little freedom to make their own decisions and exercise control over their nursing tasks. Nurses have traditionally been labeled as doctors’ subordinates, ancillary to physicians’ lead in delivery services to patients [78]. Nurses seldom question doctors’ decisions with regard to medical management [79]. A power hierarchy typically holds between the doctoring and nursing professions, with the former responsible for clinical diagnosis and the treatment of patients while the latter take in hand the care of patients, including dispensing medicines and so on.

Nowadays, the vast majority of Hong Kong nurses have a baccalaureate degree in nursing. More significantly, the model of healthcare has evolved away from one centered on the physician to one that is holistic and interdisciplinary, centered on the patient. Nevertheless, the power hierarchy seems not to have undergone little significant change over the last few decades. Nurses’ junior position in relation to doctors and their limited autonomy in professional settings may contribute to tensions or conflicts in healthcare teams [78].

As the Hong Kong population has grown, nurses have taken on an increased, and ever more complex workload, forcing them to shoulder a greater emotional burden of stress while feeling lower job satisfaction and levels of reward [14,26]. All these work-related negative contributors may impede nurses’ physical health, leading to burnout and a higher risk of psychiatric disorders [14,80,81]. Interestingly, at present, very little research has sought to disentangle the association between job satisfaction and depression in the nursing population in Asia, meaning that cross-national comparisons of our prevalence estimates with other studies were not possible.

Age and clinical experience emerged as significant correlates of stress in our multivariate analysis. Our study suggests that younger, more inexperienced nurses, particularly those between 25 and 34 years old, and with fewer than ten years of clinical experience, seem to experience more frequent symptoms of stress than older experienced nurses. In other words, age and clinical experience form an inverse relationship with the severity of symptoms of stress. Our findings match those of Yoon and Kim [14] when they suggest that younger, inexperienced nurses tend to exhibit more psychiatric symptoms than their older, experienced counterparts.

Newly qualified nurses may experience acute anxiety, stress or psychological disturbances in the course of the transition from being a student to a qualified nurse. They may experience self-doubt, feelings of inadequacy and lower self-esteem when confronted with complex clinical situations. Younger nurses may also lack the clinical experience to deal with complex cases on their own and thus may be more susceptible to experience work-related stress, workplace violence, bullying or emotional abuse in interactions at work. These younger nurses may come to feel more isolated or stressed if they perceive a lack of support from senior managers or clinical supervisors, or even from colleagues and peers.

Cumulative stress may lead to physiological arousal impeding nurses’ capacity to deal with complex tasks. Nurses under a persistently high state of arousal may feel burned out, frustrated, irritated and exhausted; they may have difficulty sleeping or succumb to depression [53,82]. There is thus a need for effective stress-coping mechanisms to protect nurses from psychiatric morbidity.

Interestingly, we found that drinking alcohol was a significant correlate of stress in the final model. No more than 20% of our respondents reported drinking, and even then at an incidence of no more than once or twice a month; even so, these individuals were at higher risk (OR = 1.8, 95% 1.19–2.72) of reporting symptoms of stress than non-drinkers or those who drank habitually (daily to a few times per week) (OR = 1.6, 95% CI 0.75–3.52). We can think of broadly three speculative explanations: (1) stressed-out nurses drink socially to relax, an interpretation that becomes more plausible given that most nurses in our study classified themselves as physically inactive (76.5%) and not giving themselves time off for entertainment (61.7%); (2) many nurses suffer from chronic physical and psychiatric illnesses, which inevitably impair their ability to adopt a healthy lifestyle (e.g., taking exercise, engaging in some form of entertainment or hobbies), meaning in consequence they turn to drink as a stress-reliever, and (3) nurses drank to help them overcome stress and get to sleep.

As cited by some authors, it is not uncommon for nurses to have recourse to coping strategies that may harm their health (e.g., smoking, taking drugs, drinking alcohol) [83] or over-eating [84]. Some studies come to a similar conclusion as the present study in finding a bi-directional relationship between alcohol consumption and stress [85]. Current drinkers are more likely to be experiencing symptoms of stress in that they drink to cope with stressful situations in their life [86].

Nevertheless, it would be unfair to suggest that most nurses in our study are using negative coping strategies to get to grips with work-related stress. Older research like that of Callaghan *et al.* [67] examines nurses’ coping strategies for 500 Hong Kong nurses, finding that most seek to deal with difficulties by soliciting the support of their friends and colleagues. Cognitive management (not thinking about work after work), engagement in other leisure activities (e.g., resting, sleeping well, relaxation) and finding spiritual comfort were also found useful.

Our findings are mostly consistent with other published works and offer a degree of insight into nurses’ mental health in Hong Kong. Risk factors associated with depression, anxiety and symptoms of stress in our study were also similar with other research. At present, most research have placed strong emphasis on examining depression/stress or depression and stress but rarely investigated anxiety and its associated factors in nurses. This also explained why we used DASS 21 to measure three dimensions of psychiatric morbidity in our study instead of using traditional psychological instrument (e.g., CES-D) to measure only one dimension of depression. More significantly, we brought forward a significant message that nurse professionals either locally, or globally, are not immune to mental health issues. Mental health research and prevention may thus need to address the specific mental health needs (e.g., barriers of professional help-seeking) of healthcare professionals across the globe to restore optimal wellness among the healthcare workforce.

## 4. Implications of This Study

### 4.1. Health Authority Level

The study’s findings would suggest the HK healthcare authority and HKSAR government should take a proactive role in working to improve nurses’ wellbeing, making professional counseling, psychiatric consultation and other forms of psychological support more widely available in a more transparent way. Healthcare providers should also aim to foster a positive culture that encourages staff to discuss their psychological or psychiatric symptoms openly without fear of stigma, embarrassment, denigration or discrimination.

### 4.2. Managerial Level

We recommend that hospital managers and nurses’ more direct managers should strive to create a stress-free working environment where work practices promote nurses’ mental wellbeing or, at least, are configured to minimize the deleterious effects of stress on nurses’ professional functioning. With support from managers, nurses may be more willing to consult experts like psychologists, psychiatrists and mental health counselors so that any emergent disorders may be detected and treated early.

### 4.3. Educational

Education on stress management, cognitive-behavioral therapy, mindfulness-based interventions, meditation, mental health (perhaps as taught through workshops or awareness programmes), therapeutic lifestyle models, and suicide prevention, as well as on related topics, may help to inculcate resilience in nurses and strengthen their skills in dealing with stress.

### 4.4. Individual Level

Nurses themselves should cultivate heightened awareness of their mental health, reviewing themselves periodically to keep track of any increases in depression, anxiety or symptoms of stress. They should be familiar with all resources available in their work and home communities to address any problems and maintain themselves in a state of the best possible mental wellness.

## 5. Conclusions

Our study successfully identifies significant predictors of these forms of psychosocial disturbance in nurses. Risk factors include socio-demographic characteristics such as age and marital status; individual factors like chronic illness, sleep problems and self-perceived mental health; lifestyle factors include exercise and time for leisure and work-related factors, including clinical specialty, upset with colleagues disturbance, and job satisfaction.

Lifestyle factors emerged as significant contributors to poor mental health. The implication is that nurses should make changes, if necessary, to their lifestyle to try to ensure a good work-life balance and safeguard their functioning at work and personal well-being. In validating and deepening the current study findings, it may be helpful to conduct in-depth focus group interviews to begin to tease out causal relationships we hypothesize between psychiatric symptoms and personal and professional factors. This will make a start on formulating effective strategies for promoting nurses’ mental balance and restoring them to health.
